# Human Plasma Metabolomics Implicates Modified 9-cis-Retinoic Acid in the Phenotype of Left Main Artery Lesions in Acute ST-Segment Elevated Myocardial Infarction

**DOI:** 10.1038/s41598-018-30219-w

**Published:** 2018-08-28

**Authors:** Lei Huang, Lei Zhang, Tong Li, Ying-wu Liu, Yu Wang, Bo-jiang Liu

**Affiliations:** 1 Heart Center, Tianjin Third Central Hospital, Tianjin, P.R. China; 2 Tianjin Institute of Hepatobiliary Disease, Tianjin, P.R. China; 3 Artificial Cell Engineering Technology Research Center of Public Health Ministry, Tianjin, P.R. China; 4 Department of Clinical Laboratory, Tianjin Third Central Hospital, Tianjin, P.R. China

## Abstract

The detection of left main coronary artery disease (LMCAD) is crucial before ST-segment elevated myocardial infarction (STEMI) or sudden cardiac death. The aim of this study was to identify characteristic metabolite modifications in the LMCAD phenotype, using the metabolomics technique. Metabolic profiles were generated based on ultra-performance liquid chromatography and mass spectrometry, combined with multivariate statistical analysis. Plasma samples were collected prospectively from a propensity-score matched cohort including 44 STEMI patients (22 consecutive LMCAD and 22 non-LMCAD), and 22 healthy controls. A comprehensive metabolomics data analysis was performed with Metaboanalyst 3.0 version. The retinol metabolism pathway was shown to have the strongest discriminative power for the LMCAD phenotype. According to biomarker analysis through receiver-operating characteristic curves, 9-cis-retinoic acid (9cRA) dominated the first page of biomarkers, with area under the curve (AUC) value 0.888. Next highest were a biomarker panel consisting of 9cRA, dehydrophytosphingosine, 1H-Indole-3-carboxaldehyde, and another seven variants of lysophosphatidylcholines, exhibiting the highest AUC (0.933). These novel data propose that the retinol metabolism pathway was the strongest differential pathway for the LMCAD phenotype. 9cRA was the most critical biomarker of LMCAD, and a ten-metabolite plasma biomarker panel, in which 9cRA remained the weightiest, may help develop a potent predictive model for LMCAD in clinic.

## Introduction

Coronary artery disease (CAD) is the leading cause of mortality and morbidity worldwide^1^. CAD presents as various phenotypes, including variations in the number of affected vessels, location of lesions, and degree of vascular stenosis. These variations may suggest differing mechanisms of atherosclerosis. Among the different anatomic phenotype variants, severe left main coronary artery disease (LMCAD) accounts for 3% to 10% of patients undergoing coronary angiography. It is the highest-risk lesion subset, and correlates with worse prognosis following heart attack, compared with non-LMCAD^2^. The LMCAD contributes more than 75% of the blood supply to left ventricular cardiomyocytes in right-dominant or balanced type coronary circulation, and 100% in left dominant type. Therefore, severe LMCAD decreases flow to a majority of the myocardium, predisposing the patient to fatal cardiovascular events, e.g. refractory cardiogenic shock and malignant arrhythmia^3^. The pathogenesis of LMCAD has not yet been clearly elucidated. A strong genetic component was proposed a decade ago on account of observation of familial aggregation of LMCAD^4^. Subsequently, conflicting results have emerged regarding the relationship between the phenotype and genetic susceptibility^5–9^. Thus, further study is needed to explore the pathogenesis of this pathology.

Metabolomics is a biosystematic research method that detects alterations *in vivo* when certain stimuli are introduced. The approach focuses on the change of end-products within a biological system affected simultaneously by distinct genotypes and environments^10^. Some technologies such as ultra-performance liquid chromatography and mass spectrometry (UPLC/MS) assist diagnosis of a disease or may help monitor its progression, all from a body fluid sample. The technology promotes a more comprehensive, real-time understanding of disease evolution^11^. In a previous study, using UPLC/MS, we showed that sphingolipid metabolism was the most altered pathway in young ST-elevated myocardial infarction (STEMI) patients, and may represent a valuable prognostic factor or potential therapeutic target^12^.

Due to its important clinical significance but insufficient volume of research, the pathogenesis of LMCAD has been attracting increasing attention. To the best of our knowledge, there is a gap in the fund of knowledge that metabolomics can contribute to the study aimed at LMCAD. The aim of this study was to identify plasma characteristic metabolite modifications, and to discover potential biomarkers with good discriminative capability for the LMCAD phenotype. A flow chart illustrating the study design is shown in Supplementary Fig. S1.

## Results

### Baseline characteristics in the unmatched and propensity-matched groups

During the study period, 462 STEMI patients were recruited. 227 patients were eligible for the study, including twenty-two LMCAD and 205 non-LMCAD patients. A one-to-one propensity score matching (PSM) created twenty-two pairs.

Table 1 shows the comparisons of baseline characteristics between LMCAD and non-LMCAD groups before and after PSM, respectively. Before PSM, the LMCAD group was shown to be older, had higher Gensini Scores, higher peak values of myocardial enzymes, higher incidence of multiple-vessel involvement, and higher IABP utilization. Nevertheless, all of the baseline characteristics were well balanced after PSM.

**Table 1:** Comparison of baseline characteristics between LMCAD and non-LMCAD groups.

Parameters	Pre-matched Groups			Post-matched Groups		
	LMCAD (n = 22)	Non-LMCAD (n = 205)	*p*	LMCAD (n = 22)	Non-LMCAD (n = 22)	*p*
Age	67 (60,79)	61.0 (55,71)	0.031	67.4 ± 12.2	64.1 ± 12.4	0.369
Male n (%)	17 (77.3)	154 (75.1)	0.824	17 (77.3)	18 (81.8)	1.000
BMI (Kg/m^2^)	23.2 (21.4,26.7)	24.8 (22.5,27.0)	0.269	24.4 ± 3.8	23.6 ± 3.3	0.433
Killip			0.302			0.306
I	17 (77.3)	177 (86.3)		17 (77.3)	11 (50.0)	
II	2 (9.1)	19 (9.3)		2 (9.1)	4 (18.2)	
III	2 (9.1)	5 (2.4)		2 (9.1)	4 (18.2)	
IV	1 (4.5)	4 (2.0)		1 (4.5)	3 (13.6)	
S2B (h)	4.4 (3.5,5.9)	4.8 (3.6,7.7)	0.344	4.4 (3.5,5.9)	5.1 (4.0,5.8)	0.205
**Comorbidity**						
Hypertension n (%)	11 (50)	107 (52.2)	0.845	11 (50)	10 (45.5)	1.000
Diabetes n (%)	3 (13.6)	52 (25.4)	0.222	4 (18.2)	5 (22.7)	1.000
Stroke n (%)	2 (9.1)	20 (9.8)	0.920	2 (9.1)	4 (18.2)	0.664
Gensini Score	82.0 (55.8,129.5)	54 (36,80)	<0.0001	89.6 ± 37.5	71.8 ± 32.0	0.097
Culprit vessel n (%)			<0.001			0.140
LAD	9 (40.9)	107 (52.2)		9 (40.9)	14 (63.6)	
Lcx	1 (4.5)	22 (10.7)		1 (4.5)	0 (0)	
RCA	10 (45.5)	76 (37.1)		10 (45.5)	8 (36.4)	
LM	2 (9.1)	0 (0)		2 (9.1)	0 (0)	
Vessels involved No.			0.013			0.09
1	2 (1.1)	45 (21.9)		2 (9.1)	6 (27.3)	
2	3 (13.6)	69 (33.7)		3 (13.6)	6 (27.3)	
3	17 (77.3)	91 (44.4)		17 (77.3)	10 (45.5)	
CKMB peak value (U/L)	286 (147,464)	170 (95,285)	0.030	286 (147,464)	143 (87,276)	0.116
IABP n (%)	4 (18.2)	7 (3.4)	0.002	4 (18.2)	3 (13.6)	1.000

Abbreviations: S2B sympotm to Balloon; LAD left anterior descending artery; Lcx eftcircumflex artery; RCA right coronary artery; LM left main artery; CKMB creatine kinase isoenzyme-MB; IABP intraaortic balloon pump.

### Discriminant analyses to distinguish LMCAD and control groups

**Figure 1:**
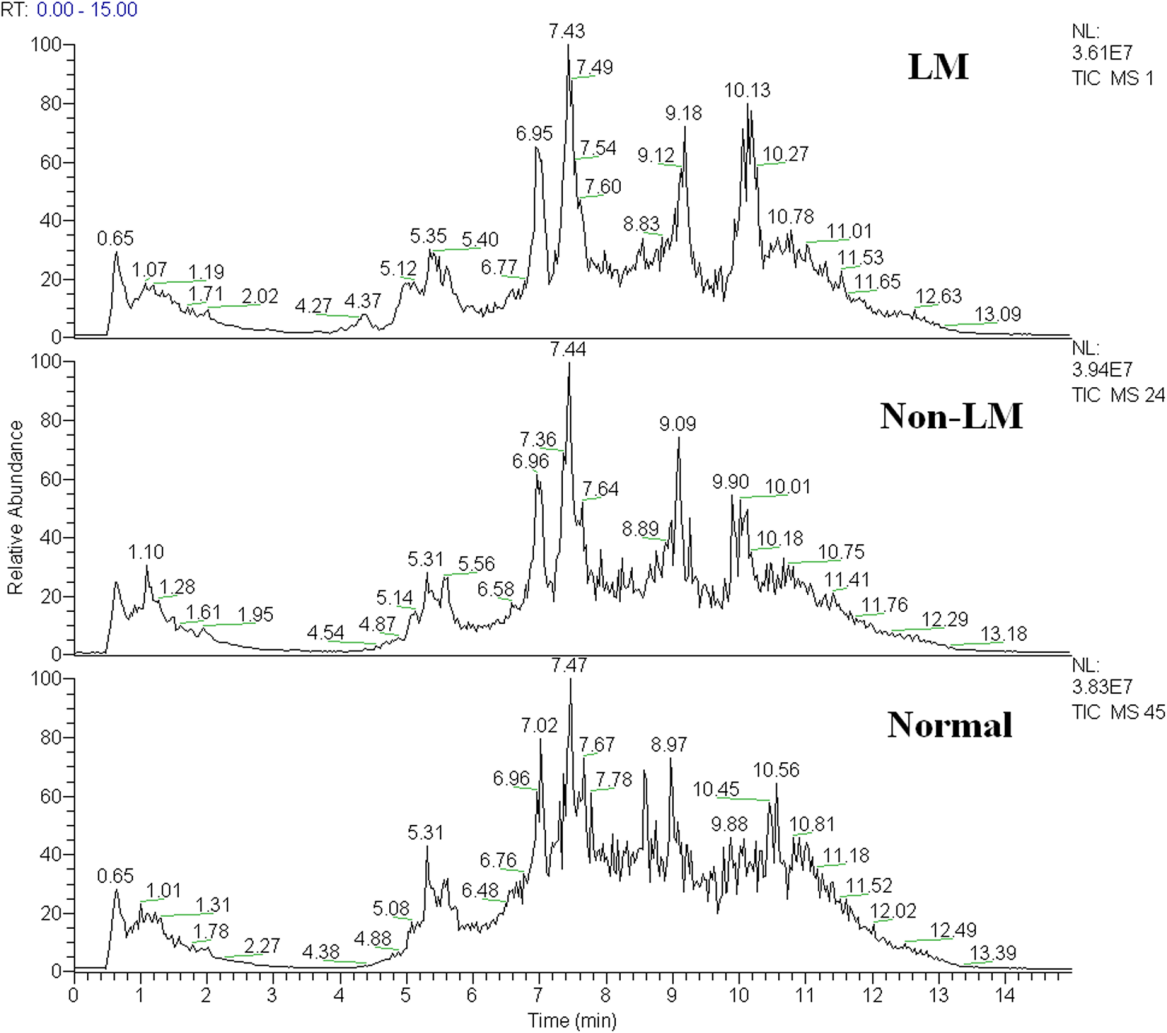
Total ion chromatogram of metabolic profiles in various groups which was obtained from SIMCA-P 12.0 (one sample chosen randomly). LM: group with left main coronary artery disease; non-LM: group without left main coronary artery disease.

**Figure 2:**
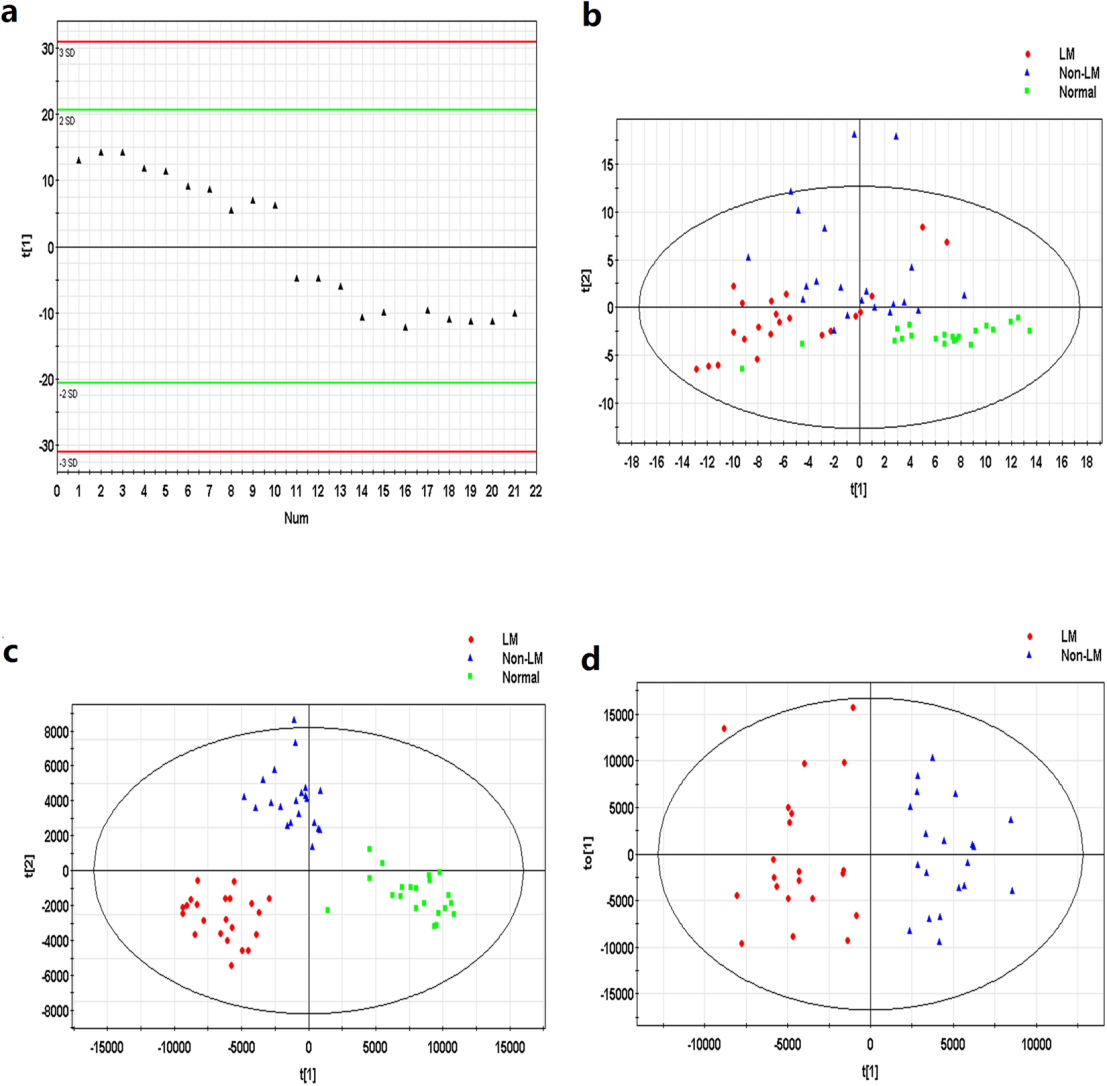
(**a**) Score plot of principal component (t[1]) in the PCA model for QC samples. (**b**) PCA score plot of LMCAD group (LM), non-LMCAD (non-LM) and healthy subject group (normal). (**c**) OPLS-DA score plot of LMCAD (LM), non-LMCAD (non-LM) and healthy control (normal) groups. Two predictive principal components and six orthogonal principal components (R2X = 76.3%, R2Y = 86.2%, Q2 = 56.8%). (**d**) OPLS-DA score plot of the LMCAD (LM) and non-LMCAD (non-LM) group. One predictive principal components and two orthogonal principal components (R2X = 43.2%, R2Y = 82.7%, Q2 = 58.9%). Notes: All figures were derived from SIMCA-P 12.0 and every point in the figure represents a sample. R2 scores indicate model performance, and Q2 scores estimate reproducibility on the basis of cross-validation.

Typical total ion current chromatogram from a sample in UPLC/MS is shown in Fig. 1, where substantial alterations can be observed between the two chromatograms. Multivariate analysis of the quality control (QC) samples indicated the peak area deviation was below two times a standard deviation, confirming that the analytical results were reliable (Fig. 2a). After data pretreatment and standardization, a principal component analysis (PCA) model was established with seven principal components (R2X = 50.9%, Q2 = 16.0%) for all participants (LMCAD, non-LMCAD and healthy control). The score plot of its first two principal components is shown in Fig. 2b, which demonstrates a trend of intergroup separation on the score plots. An orthogonal partial least squares-discriminant analysis (OPLS-DA) model was then used to magnify the nuances. The resulting model developed a better separation into each cluster (Fig. 2c,d), as well as in permutation tests (999 times) (*p* < 0.05).

### Comparison of characteristic metabolites among groups

Fourteen metabolites contributing significantly to the separation between LMCAD and non-LMCAD group were identified from a two-component OPLS-DA model of the UPLC/MS spectral data sets (Table 2).

**Table 2:** Metabolite identification results and the difference between interested groups.

m/z	RT^a^ (min)	Metabolite	Metabolic pathway	VIP^b^	*p-*Value^c^	FC^d^	LM vs. non-LM^‡^	LM vs. Control^§^	Non-LM vs. Control^§^
520.34	7.00933	LysoPC (18:2 (9Z,12Z))	Phospholipid metabolism	6.00	<0.0001	0.56	Down^†^	Down^†^	Down^†^
522.356	7.61744	LysoPC (18:1 (9Z))	Phospholipid metabolism	4.51	<0.0001	0.57	Down^†^	Down^†^	Down^†^
544.34	6.96251	LysoPC (20:4 (5Z,8Z,11Z,14Z))	Phospholipid metabolism	3.43	<0.0001	0.58	Down^†^	Down^†^	—
568.34	6.91188	LysoPC (22:6 (4Z,7Z,10Z,13Z,16Z,19Z))	Phospholipid metabolism	2.39	<0.0001	0.50	Down^†^	—	Up*
318.24	7.29255	9-cis-Retinoic acid	Retinol metabolism	2.65	0.00014	2.75	Up^†^	Up^†^	Up^†^
546.355	7.27006	LysoPC (20:3 (5Z,8Z,11Z))	Phospholipid metabolism	1.98	0.00028	0.51	Down^†^	Down^†^	—
494.324	6.78781	LysoPC (16:1 (9Z))	Phospholipid metabolism	1.91	0.00053	0.47	Down^†^	Down^†^	Down^†^
569.314	4.35167	Protoporphyrinogen IX	Porphyrin and chlorophyll metabolism	1.69	0.00063	1.01	—	Up^†^	Up^†^
146.059	1.94401	1H-Indole-3-carboxaldehyde	Porphyrin and chlorophyll metabolism	1.56	0.00075	0.66	Down^†^	—	Up^†^
510.355	8.02127	LysoPC (17:0)	Phospholipid metabolism	1.27	0.0010	0.44	Down^†^	Down^†^	Down^†^
338.267	6.30823	Dehydrophytosphingosine	Phospholipid metabolism	1.13	0.0052	1.23	Up^†^	Up^†^	Up^†^
457.232	6.80351	LPA (18:2 (9Z,12Z)/0:0)	Phospholipid metabolism	1.09	0.010	0.59	Down^†^	Down^†^	Down^†^
518.326	6.63885	LysoPC (18:3 (9Z,12Z,15Z))	Phospholipid metabolism	1.06	0.019	0.39	Down^†^	Down^†^	Down^†^
506.36	8.92995	LysoPC (P-18:1 (9Z))	Phospholipid metabolism	1.02	0.034	0.95	—	—	Down^†^

*p < 0.05; ^†^*p* < 0.01; ^‡^Compared with non-LMCAD group, ^§^Compared with control group. –: No significant difference. Abbreviations: LysoPC lysophosphatidylcholines; LPA lysophosphatidic acid. ^a^Retention Time. ^b^Variable Importance in Projection. Values (cut-off threshold: 1.0) were obtained from the OPLS-DA model. ^c^*p*-Values were calculated using the Student’s t-test. ^d^FC, fold change. Values > 1 indicate that levels were higher in LMCAD group than non-LMCAD group; values <1 indicate that levels were lower in LMCAD group compared to non-LMCAD group.

### Metabolic pathways and functional analysis

**Figure 3:**
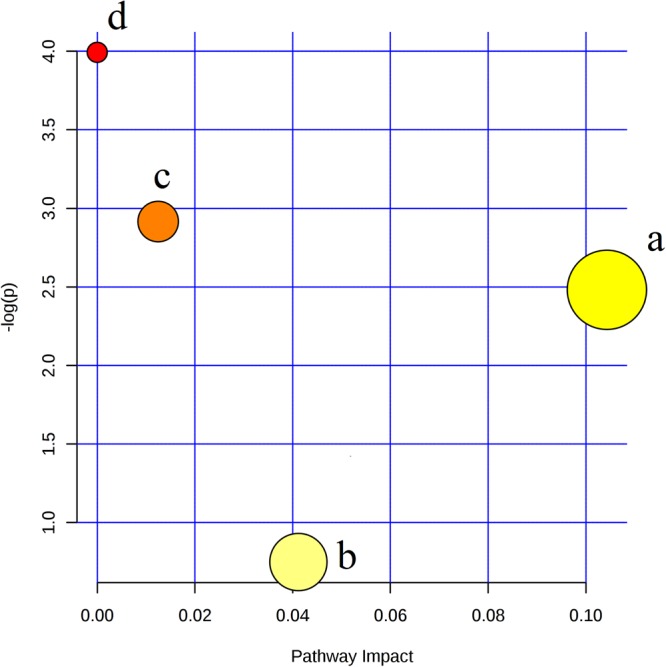
Pathway analysis summaries from MetaboAnalyst 3.0. All the matched pathways are displayed as circles in metabolome view. (**a**) Glycerophospholipid metabolism, (**b**) Porphyrin and chlorophyll metabolism, (**c**) Glycerolipid metabolism, (**d**) Retinol metabolism.

Further metabolic pathway analysis illustrated that the metabolites identified for LMCAD discrimination were responsible for the metabolism of glycerophospholipid, retinol, glycerolipid, porphyrin and chlorophyll. Importantly, the retinol metabolism pathway was shown to possess the strongest discriminative power for LMCAD phenotype despite the fact that the glycerophospholipid metabolism pathway was shown to be the most altered pathway (Fig. 3 and Supplementary Table S1).

### Biomarker identification and performance evaluation

**Figure 4:**
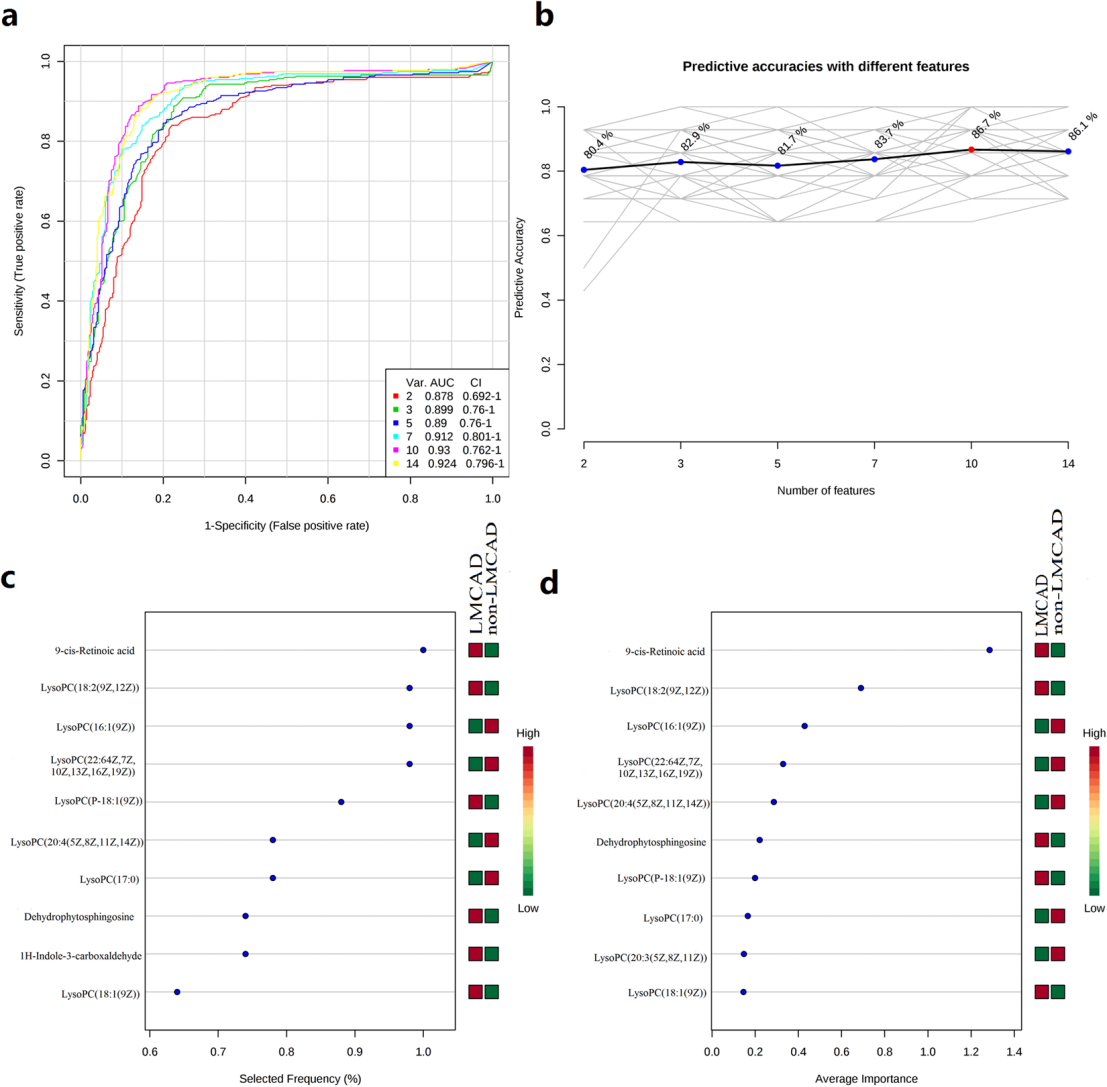
The process of feature selection, model building, and performance evaluation performed multiple times through Monte-Carlo cross validation via MetaboAnalyst 3.0 (**a**) ROC curves of all models based on the cross validation performance. Model 5 (with 10 features) gives the largest AUC and (**b**) the highest predictive accuracy. Ten significant features ranked based their frequencies of being selected (**c**) and average importance (**d**) during cross validation.

Univariate analysis for biomarker identification using receiver operating characteristic (ROC) curve demonstrated that 9-cis-retinoic acid (9cRA) dominated the first page of biomarkers, with area under the curve (AUC) value 0.888. The corresponding optimal cutoff (after log transformation) with associated sensitivity and specificity was −0.0709 (0.8, 0.9, Supplementary Table S2). A multivariate ROC curve based exploratory analysis was then performed for the purpose of feature selection, model building, and performance evaluation. The Linear Support Vector Machine (SVM) for the classification and “SVM built-in” for the feature ranking method was generated as a multivariate algorithm to perform biomarker identification. The ROC curves from all models based on cross validation performance indicated that Model 5 entered 10 features, including 9cRA, dehydrophytosphingosine, 1H-Indole-3-carboxaldehyde and seven variants of lysophosphatidylcholines (LysoPCs). This model had the largest AUC (0.933) and highest predictive accuracy (89%) (Fig. 4a,b). In the biomarker-predicting model building and performance evaluation based on Monte Carlo cross-validation, 9cRA was shown to have the greatest chance to appear in the predictive biomarker panel with highest average importance (Fig. 4c,d), suggesting it was the most important biomarker in our biomarker panel.

## Discussion

Severe LMCAD is the most dangerous subtype of coronary disease. It correlates with worse clinical outcomes than those of non-LMCAD in various anatomic types of obstructive coronary artery disease. The clinical manifestations of the phenotype are diverse, ranging from asymptomatic to stable angina, to acute coronary syndrome and heart failure. Early diagnosis of the entity using both invasive and noninvasive techniques is challenging. However, few studies have focused on the pathogenesis, and early reliable biomarkers have not yet been discovered. This may be because LMCA is not traditionally considered a risk site for atherosclerotic plaque formation. Thus, deeper insight into metabolic mechanisms related to LMCAD should be acquired and biological importance of potential biomarkers should be evaluated.

The identification of novel biomarkers for use in assessing early risk of LMCAD have potentially important clinical implications, such as identifying high-risk individuals, and adapting appropriate therapeutic management. LMCAD was initially found to be inherited and frequently shared by siblings with CAD^4^. Thereafter, many genes have been demonstrated partly to contribute to severe LMCAD pathogenesis, including genes associated with the systems of interleukin^5^, inflammation^6^ and prostaglandin synthetase^7^. Individuals with certain polymorphisms of these genes are susceptible to LMCAD. However, certain polymorphisms in the coding genes of important pathway proteins involved in atherosclerosis, e.g. LOXIN (a cell receptor for oxidized low-density lipoproteins)^8^, and cholesteryl ester transfer protein^9^, were not shown to associate with susceptibility to the LMCAD phenotype. Genes alone do not predict atherosclerosis, because of the interaction of numerous genes and environmental influences. Genomics merely indicates the potential causes for a phenotypic response, but it cannot predict what will happen at the next level. Metabolomics provides a functional view of an organism as determined by the sum of its genes, RNA, proteins, and environmental factors (including nutrition and medications). For these reasons, metabolomics, as the youngest ‘omics’ disciplines, might become the most promising technique to solve the problem.

Against this background, we explored the usefulness of UPLC/MS, a high-throughput metabonomic technology to analyze differentially expressed metabolites that might, for the first time, allow identification of possible biological processes in LMCAD and potentially valuable predictive biomarkers. We compared the plasma samples of LMCAD, non-LMCAD, and healthy control groups. We discovered fourteen significantly altered metabolites in LMCAD patients compared to non-LMCAD patients, and predicted the major metabolite network by pattern recognition and pathway analysis. The identified target metabolites were found to encompass a variety of pathways related to glycerophospholipid/glycerolipid-related metabolism (LysoPCs, lysophosphatidic acid and dehydro-phytosphingosine), vitamin A metabolism (9cRA), porphyrin and chlorophyll metabolism (1H-Indole-3-carboxaldehyde). We suggest that one or several combinations of these metabolites maybe helpful for revealing the complex mechanism or become predictive biomarkers for LMCAD.

Since use of various selection techniques may result in different compounds being indicated as significant, accurate identification of differential metabolites between groups is a prerequisite for drawing reliable conclusions. According to the introduction of methodology from the primary literature, a common approach to identify differential metabolites is to select metabolites based on VIP > 1, complemented with intergroup *p* values or fold-changes as verification^13,14^. We employed the above-mentioned method and our results demonstrated there was great consistency between the ranking of the metabolites by VIP > 1 and the ranking by the corresponding intergroup *p* values. The fold-change of each metabolite also reflected the relationships of the corresponding peak intensities between the two groups. This confirmed that the use of VIP-value as a screening criterion was reasonable and robust. However, we also found that the central differential metabolites obtained based on the ranking of *p* values were not completely consistent with those obtained based on the VIP ranking. This may be ascribed, to a great extent, to the higher complexity of metabolite composition and substantial changes occurring between disease and control groups. Therefore, the screening results obtained by different statistical algorithms may result in heterogeneity that could be regarded as acceptable.

It should be also bore in mind that some metabolites detected in the preparation might partially originate from cell-derived microparticles (MPs) contained in plasma. This potential source has several major implications for our study. First, MPs from endothelial cells were demonstrated as markers of endothelial dysfunction and their counts highly correlated with apoptotic endothelial cell^15^. MPs in plasma have been reported to be not only significantly elevated in acute coronary syndromes compared to stable disease or controls, but also to reflect the severity of vascular lesions^16^. Second, MPs can transport and disseminate potent bioactive effectors, including procoagulant, proinflammatory or apoptogenic mediators to activate their targets, e.g. endothelial cells, and to prime pathological processes during the onset of acute coronary syndrome^17^. Third, the characterization of MPs facilitates making a reasonable explanation for the changes of lipid composition in plasma^18^.

In the biomarker-predicting model building and performance evaluation, small AUC of single one metabolite indicated relatively low diagnostic capability to the phenotype. Of these, 9cRA dominated the first page of biomarkers, with AUC value 0.888. However, a biomarker panel containing ten metabolites of 9cRA, dehydrophytosphingosine, 1H-Indole-3-carboxaldehyde and seven variants of LysoPCs displayed the highest AUC (0.933). After systematic metabolic pathway analysis combined with multivariate statistical analysis, we found the homeostasis of retinol metabolism pathway mediated regulation of LMCAD pattern as a crucial mechanism. 9cRA, again, was indicated to have the greatest chance to appear in the predictive biomarker panel with highest average importance. These metabolic alterations and the associated pathways provide new insights into the pathogenesis of LMCAD. Considering its remarkable diagnostic capability, the novel biomarker panel might lead to new strategies for prediction and treatment of LMCAD.

As we expected, glycerophospholipid metabolism pathway was shown to be the top altered pathway. Some significantly perturbed metabolites, e.g. LysoPCs, composed the highest proportion of the characteristic metabolites. The concentrations of lysophosphatidic acid and multiple kinds of LysoPCs were noted to be significantly down-regulated and sphingosines significantly up-regulated in STEMI compared to the PSM healthy controls. These findings are consistent with our previous findings of metabolic profile analyses from STEMI patients by means of UPLC/MS technology^12^. The physiological function of glycerophospholipids mainly includes the following: first, mediation of many cell-signaling pathways in monocytes/macrophages^19,20^ and specific receptors^21^, particularly participation in inflammatory responses; second, these metabolites correlate well with the main components of cell membrane, and therefore to cell proliferation, differentiation, apoptosis, and biological energy supply^12,22^. Additionally, a further significant concentration reduction of LysoPCs, lysophosphatidic acid and increase of dehydrophytosphingosine in the LMCAD group might be responsible for the LMCAD group suffering more severe myocardial necrosis than the non-LMCAD group after STEMI episode. This assumption was suggested by the significantly higher peak value of myocardial enzymes in the LMCAD group compared to that of the non-LMCAD group.

The most important conclusion from our data is that retinol metabolism dyshomeostasis is substantially associated with the LMCAD phenotype. 9cRA, a member of structurally simple lipid molecules derived from retinol, was demonstrated to have the best discriminative performance for this phenotype. In the biomarker-predicting model building and performance evaluation based on Monte Carlo cross-validation, 9cRA was found to be selected with almost 100% of the time in the model via the SVM feature selection algorithm. This was superior to the other nine differential metabolites. 9cRA, a key component of this metabolic pathway, trans-activates numerous genes and exerted pleiotropic effects on cellular growth, differentiation and immune response in all vertebrates^23,24^. 9cRA is generated from retinaldehyde by Raldh and metabolize into inactive derivatives by Cyp26^25^. Contrarily, the Dhrs enzyme family converts retinaldehyde back to retinol, thereby regulating the amount of available substrate for 9cRA production. Thus, a complex feedback system is generated from enzymes and substrates to maintain physiological homeostasis. Subtle disturbances of retinoic acid levels can have detrimental effects on prenatal developmental defects, and is responsible for a variety of pathological conditions after birth. This has been shown in extensive animal studies as well as in human subjects^26,27^.

In our study, we found that the concentration of 9cRA in the LMCAD group was significantly higher than that of the non-LMCAD group. The imbalance of retinoid metabolism in LMCAD patients could be explained by the oxidation theory of lipids and their importance in atherosclerosis, considering that 9cRA is an oxidized metabolite of the fat-soluble vitamin A. It is well known that atherosclerosis, i.e., luminal stenosis of arteries under oxidative stress, is related to oxidative changes of low density lipoproteins (LDL). Oxidation of LDL produces lipid peroxidation products such as lipid peroxides that in turn influence the metabolism of fat-soluble vitamins inside cells. There is growing evidence that increased intake of antioxidants vitamins, e.g. vitamin A and beta-carotene, have a protective role in coronary heart disease by inhibiting lipid peroxidation^28^. Low intake of vitamin A and beta-carotene, however, were found to be among of the risk factors for atherosclerosis in patients with high-homocysteine by logistic regression analysis in a recent observational study^29^. These findings confirmed a role of antioxidant compounds of vitamin A and beta-carotene against oxidative damage. Unfortunately, we have no records of the patient’s diet and vitamin supplementation habits in their medical records. Improving the knowledge of these nutrients intake may guide treatment of LMCAD and assist creation of effective nutrition support strategies.

Moreover, a better understanding of the 9cRA signaling pathway, especially the interrelation between systemic 9cRA homeostasis and gene regulation network, has been developed over the past decades. 9cRA modulates the expression of its target genes by activating two classes of nuclear receptors, retinoic acid receptors (RARs) and retinoid X receptors (RXRs)^30^. The complexity and diversity of 9cRA function is expressed by its ability to activate RXRs. RXRs are ubiquitously expressed nuclear receptors, existing as obligate heterodimers for a number of receptors, e.g. peroxisome proliferator activated (PPAR), liver X (LXR), vitamin D (VDR), thyroid hormone (TR), farnesoid X (FXR), and pregnane X (PXR). RXR heterodimers regulate multiple complex cellular processes (Supplementary Fig. 2). Any perturbation in the expression and activity of RXRs substantially influences development, metabolic diseases, cancer and atherosclerosis^31–33^.

As a subtype of atherosclerotic disease, one dysfunctional metabolic pathway highly correlated with LMCAD may be abnormal activation of macrophages into foam cells in LMCA. PPARγ, a key heterodimer partner with RXRs, was shown to participate in the regulation of cholesterol metabolism and exert protective effects against atherosclerosis in humans, mice and rabbits^34–36^. These results suggest that the nuclear receptors may serve as downstream target molecules of 9cRA, and may participate in the regulation of LMCAD phenotype. Related mechanisms may involve multiple general anti-inflammatory and potential protective effects on macrophage activation and foam cell formation, such as transcriptional interference with proinflammatory transcription factors and inhibitory effects on the production of matrix metalloproteinases, vascular cell adhesion molecule, and intercellular adhesion molecules^37–41^. Hence, 9cRA–RXR-PPARγ axial patterning, when perturbed, may contribute to the pathogenesis of LMCAD, based upon our data. A significant increase in the concentration of 9cRA in our LMCAD group may involve one or more of the following mechanisms: (1) altered levels of expression or activity of enzymes closely related to retinol metabolism pathway and consequently impaired normal feedback to regulate 9cRA biosynthesis and catabolism; (2) differential expression levels of 9-cis RA receptor-RXR and PPARγ between LMCA and other segments of coronary arteries; (3) both factors may obtain simultaneously. Further studies should assess the relative contribution of these factors genetically and epigenetically, via *in vivo* and *in vitro* studies, respectively. Clarifying these questions would provide a more valuable elucidation of the relationship between metabolites and pathological mechanisms as well as therapeutic strategies.

## Methods

### Chemical reagents and instruments

All solvents were high performance liquid chromatography grade and were used without modification. All standard compounds were purchased from Sigma-Aldrich (St. Louis, MO, USA). Formic acid, methanol and acetonitrile were obtained from Merck (Merck KGaA, Darmstadt, Germany). Distilled water was produced by a Milli-Q reagent-grade water system (Millipore; Billerica, MA, USA). UPLC was performed on a Thermo Fisher Accela system and mass spectrometry was performed on an LTQ Orbitrap XL hybrid mass spectrometer (both from Thermo Fisher Scientific, Franklin, MA, United States).

### Subjects

**Figure 5:**
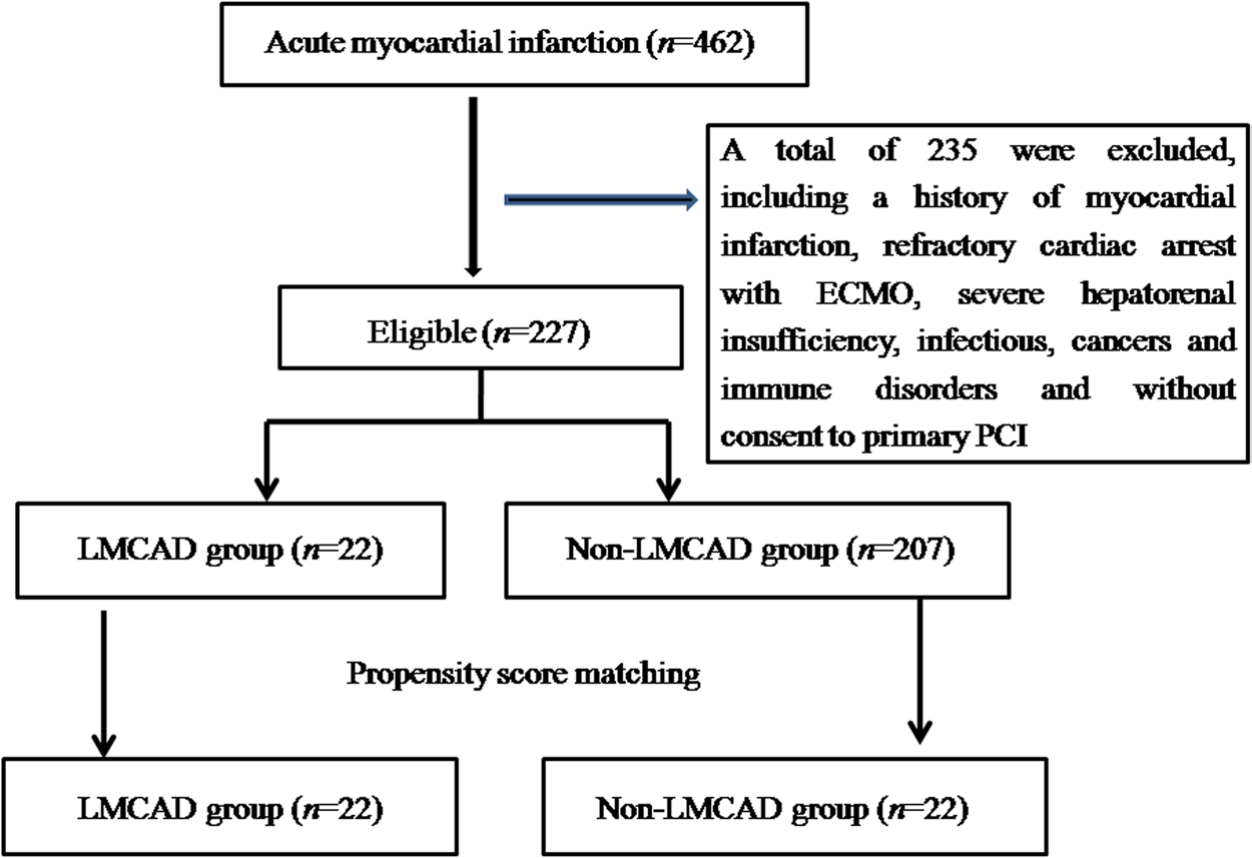
Patient selection. LMCAD left main coronary artery disease; PCI, percutaneous coronary intervention; ECMO extracorporeal membrane oxygenation.

To ensure homogeneity for all clinical features except LMCAD phenotype, a PSM non-LMCAD STEMI group (disease control) was selected. Sample size estimation was made via the MetaboAnalyst tool, based on this pilot data set. We calculated that approximately 20 samples per group would afford the study robustness (around 0.95) (enter 200 as the maximum sample size per group; leave the FDR cutoff as 0.1, Supplementary Fig. S3). Our institute is a tertiary university hospital with a primary percutaneous coronary intervention (PCI) volume for STEMI of approximately 350 cases annually. Based on our predefined entry criteria, we recruited inpatients and healthy volunteers for sixteen months (January 1 2016 to May 31 2017), totaling 22 LMCAD, 22 matched non-LMCAD STEMI patients, and 22 healthy controls. The definition of STEMI was established according to the third universal definition of myocardial infarction symptoms^42^, while LMCAD were defined as vascular stenosis from any of the three anatomic regions of left main coronary artery (LMCA)-ostium, mid-shaft and distal portion more than 50%^3^. The process of patient selection is shown in Fig. 5.

### Ethics statement

All samples were collected in accordance with the ethical guidelines of the 1975 Declaration of Helsinki. The ethics committee of Tianjin third central hospital approved the study protocol and all recruited individuals were required to sign consent forms.

### Propensity score methods

The aim of PSM is to eliminate selection bias and balance the baseline parameters that may potentially affect the pathogenesis of LMCAD. PSM between LMCAD and non-LMCAD STEMI group was developed according to the estimated propensity scores, which were calculated using a logistic regression model for the existence of LMCAD as a function of the following parameters: age, sex, BMI, Killip class, symptoms-to-balloon interval, comorbidities, affected vessel, number of vessels involved, peak value of myocardial enzyme, and intra-aortic balloon pump (IABP) utilization. We performed a one-to-one PSM analysis using nearest-neighbor matching within a caliper of 0.2 SD of pooled propensity scores. A patient who did not have a suitable match within the acceptable rank range was excluded from further analysis.

### Sample preparation for UPLC/MS analysis

Three milliliters venous blood was drawn from the peripheral vein immediately after patients admitted to wards and preserved in an EDTA anticoagulant tube. The blood was then centrifuged at 1600 × g for 10 min to isolate the plasma and preserved at −80 °C until analysis. Just before analysis, 100 µL samples were thawed at room temperature following by mixture with 400 µL methanol, intense vibration for 30 s, and incubation at 4 °C for 5 min to precipitate the protein. Then, the mixture was centrifuged at 15000 × g for 30 min at 4 °C. The supernatant was evaporated and reconstituted with 5% acetonitrile aqueous solution followed by filtrating through a 0.22 µm membrane and analyzed.

### Sample analysis

Chromatography was performed on an Accela system equipped with a binary solvent delivery manager and a sample manager. The analytical column was a Thermo Hypersil GOLD C18 reverse phase column. The UPLC mobile phase consisted of 0.1% formic acid aqueous solution (mobile phase A) and 95% acetonitrile with 0.1% formic acid (mobile phase B). Chromatographic elution time was 15 min per sample. The injection volume of the sample was 10 μL and a constant flow rate of 200 μL/min was maintained. The sample manager and column oven temperature were set at 4 °C and 20 °C, respectively.

The chromatographic elution gradient was initialized at 5% phase B and held for 3 min. In consecutive 10 min periods, phase B was linearly escalated to 50%, and then a rapid increase in phase B to 95% was completed within 3 min. After 4 min of maintaining the high volume of organic phase gradient, phase B was immediately reduced to 5% and this elution gradient was used to balance the analytical column for the final 4 min.

Mass spectrometry (MS) was performed on an LTQ Orbitrap XL hybrid mass spectrometer operating in the positive ion mode with an ion source voltage of 4.5 kV, a capillary voltage of 30 V, cone voltage of 150 V, desolvation temperature of 275 °C, sheath gas flow of 30 arb and assistant gas flow of 5 arb (99.999% nitrogen). Data were collected over 15 min in centroid mode over the mass-to-charge ratio (m/z) range 50–1000. MS resolution was at 100 000 (full width half maximum [FWHM]). Calibration standards (caffeine, Ultramark 1621 and MRFA) were provided by Thermo Fisher Scientific. Tandem mass spectrometric (MS/MS) analyses were carried out using collision-induced dissociation (CID) at 35% normalized collision energy. The collision gas was 99.9999% helium.

### Quality control

A QC sample solution was prepared by mixture of equal aliquots from each sample. Ten consecutive QC samples were injected before any test samples were run, and the remaining QC samples were inserted into the sequence after every ten samples were analyzed. The sample sequence was randomly generated by Microsoft Excel’s random number generator, and cross-contamination was obviated by inserting a blank between adjacent samples.

### Data processing and analysis

Peak detection, alignment and normalization were determined by MZmine 2.0 software. The experimental data of LC/MS were reorganized into a matrix including time, ion mass (m/z) and peak intensity. The filter conditions were a chromatography peak intensity signal-to-noise ratio >30, a retention time tolerance of ±0.1 min and an m/z tolerance of ±0.01. Then the data was imported into the SIMCA-P 12.0 software package (Umetrics, Umea, Sweden). PCA and OPLS-DA models were established after mean-centring and pareto-scaling between the groups under each condition, and checked by cross-validation. Preliminary selection of characteristic metabolites was conducted based on the corresponding variable influence on projection (VIP) value and coefficient plot generated by the OPLS-DA model. Those variables with VIP > 1.0 are deemed relevant for group discrimination. VIP statistics and S-plot were used to obtain the significant variables for subsequent metabolic pathway analysis.

### Identification of the characteristic metabolites

The parameters of the apparatus were reset based on above preliminarily selected variables. A secondary mass spectrum (MS2) scan to the control solution was performed and the MS2 of these metabolites was obtained. The selected ions were preliminarily determined by comparing the accurate m/z value, retention time retrieving from the MS2 ion chromatogram with those of authentic reference standards, such as HMDB database. The others were identified as follows: (1) The retrieved substance whose m/z deviations were lower than 0.02 was considered for further identification when the ionization modes were the same as those used in the HMDB database, (2) MS2 spectra of characteristic ions were compared with theoretical fragments from preliminary results to establish that the MS2 m/z deviation was below 0.2, the top three peaks matched and at least an 80% match was obtained between the preliminary and secondary mass spectra. The resulting two-dimensional matrix, including retention time and m/z pairs, sample names and ion peak intensity, were introduced to multivariate data analysis.

### Statistics analysis

SPSS Statistics 23.0 software (IBM, Chicago, IL, USA) was applied for clinical data analysis. Normal distributed quantitative data were expressed as a mean ± standard deviation and compared via *t* test or *t′* test between two groups. Abnormally distributed quantitative data were expressed with median and inter-quartile ranges and compared with the Mann-Whitney *U* tests. The qualitative data were presented as the frequency and composition. The differences in constituent ratio between two groups were computed via Fisher’s exact test. All *p* values were two-sided and perceived as statistical significance if *p* < 0.05. Metabolite pathway, enrichment and biomarker analyses were conducted via MetaboAnalyst 3.0 version (the Wishart Research Group, Canada), an online data tool. The relevance from genomes to pathways is interpreted through the KEGG (Kyoto Encyclopedia of Genes and Genomes) system, a database resource integrated for genomic, chemical and systemic functional information^43,44^.

### Data availability

The datasets generated during and analysed during the current study are available from the corresponding author on reasonable request.

## Limitations

This study has some limitations. Our sample size was relatively small. This may be partly explained by the relatively low incidence of severe LMCAD, as well as low volume of coronary interventions in our center and to our strict inclusion criteria. Furthermore, validating our results by other types of metabonomic platforms, e.g. GC-MS and 1H-NMR, or samples (e.g. urine) might make our results more persuasive. Finally, patients at earlier stages of atherosclerosis, such as stable angina, should be recruited in future studies with the ultimate goal that biomarkers maybe applied in early clinical diagnosis.

## Conclusions

Using a UPLC/MS platform, we determined that retinol metabolism is the most significant differential pathway in pathogenesis in LMCAD. A ten-metabolite plasma biomarker panel may serve as a potent prediction model for the phenotype, in which 9cRA possess the most robust discriminative power.

## Electronic supplementary material

supplementary data
