# Supplementary material for: Human Plasma Metabolomics Implicates Modified 9-cis-Retinoic Acid in the Phenotype of Left Main Artery Lesions in Acute ST-Segment Elevated Myocardial Infarction
Source: Sci Rep. 2018 Aug 28;8:12958. doi: 10.1038/s41598-018-30219-w (PMC6113282; doi:10.1038/s41598-018-30219-w)
Supplement: Supplementary file 1 — supplementary data [file 41598_2018_30219_MOESM1_ESM.pdf]

Original article

Human Plasma Metabolomics Implicates Modified 9-cis-Retinoic Acid in the Phenotype of

Left Main Artery Lesions in Acute ST-Segment Elevated Myocardial Infarction

Lei Huang [1,2,3], Lei Zhang[2,3,4], Tong Li [1,2,3*], Ying-Wu Liu [1,2,3], Yu Wang[1,2,3], Bo-jiang Liu[1,2,3]

1 Heart Center, Tianjin Third Central Hospital, 2 Tianjin Institute of Hepatobiliary Disease, 3 Artificial Cell Engineering Technology Research Center of Public Health Ministry, Tianjin, P.R. China 4 Department of Clinical Laboratory, Tianjin Third Central Hospital

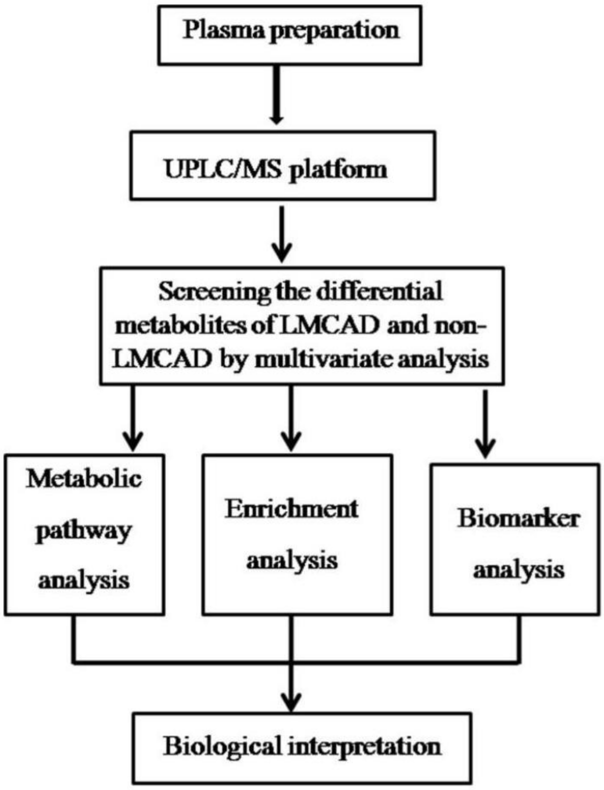

Supplementary Figure 1 Schematic flow chart of the metabolic profiling strategy used in this study. UPLC/MS, ultra-performance liquid chromatography and mass spectrometry; LMCAD, left main coronary artery disease; PCI, percutaneous coronary intervention.

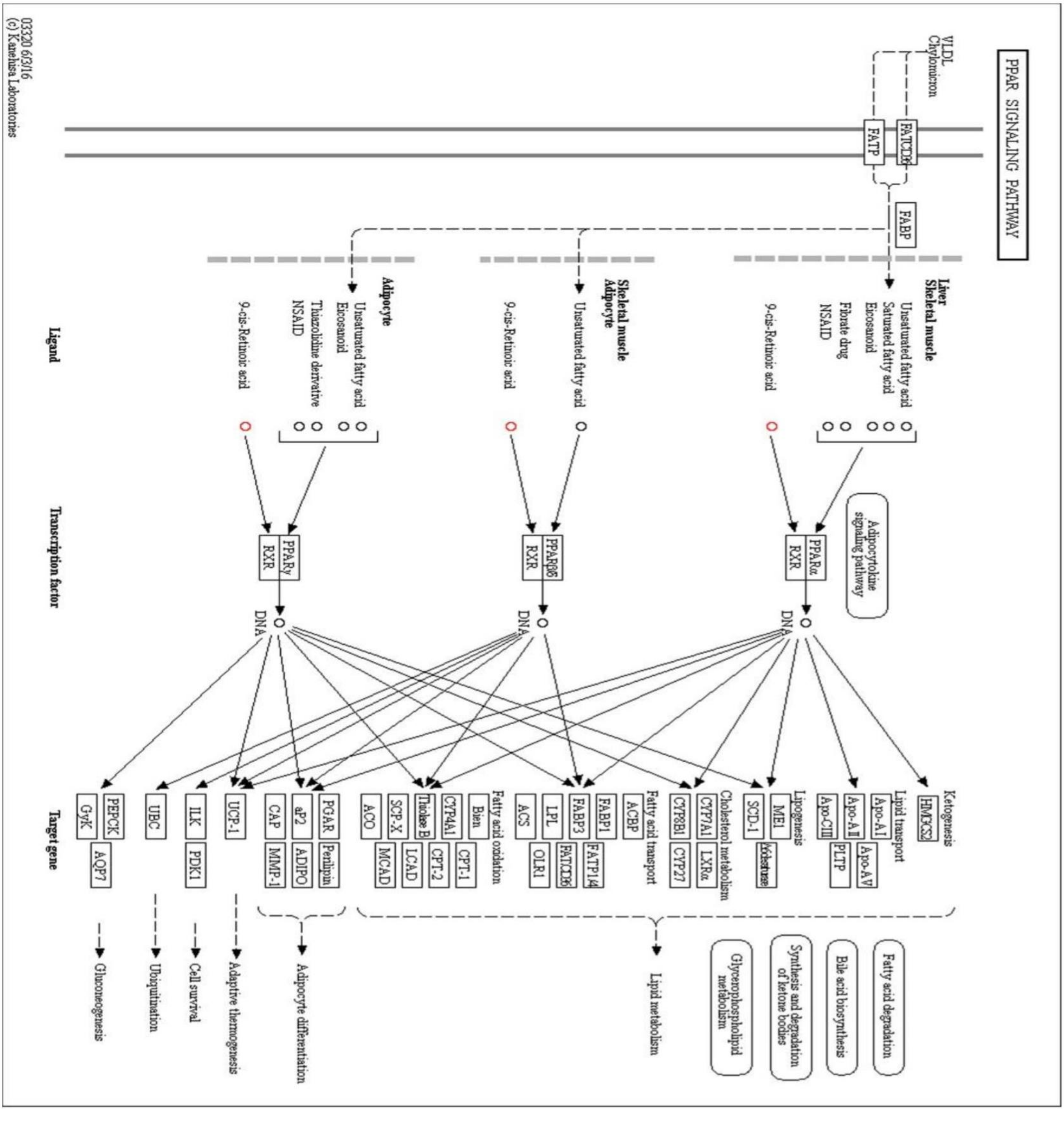

Supplementary Figure S2 Systemic analysis of PPAR signaling pathway. This map was generated from the KEGG reference map and the citation has been authorized with a written permission by the copyright holder of Kanehisa Laboratories.

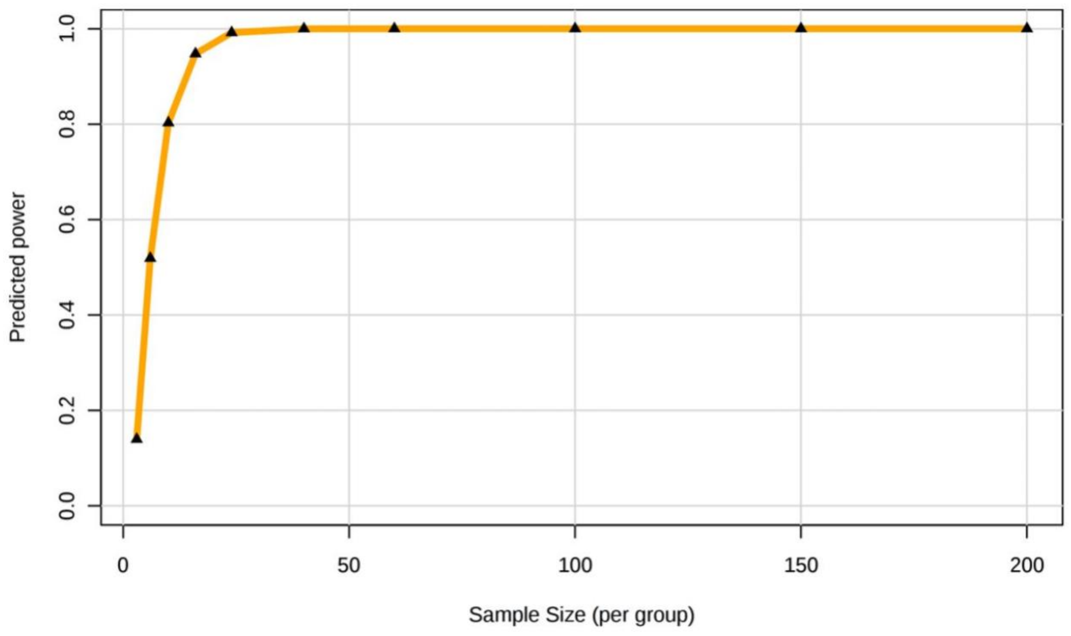

Supplementary Figure S3 Sample size estimation

Supplementary Table S1 Result from pathway analysis

Pathway name	Match Status	Raw p	-log(p)	Holm P	Impact
Glycerophospholipid metabolism	2/39	2.48E-03	5.999	0.20	0.104
Retinol metabolism	1/22	4.49E-02	3.1031	1.0	0.00
Glycerolipid metabolism	1/32	6.48E-02	2.7367	1.0	0.01
Porphyrin and chlorophyll metabolism	1/104	1.98E-01	1.6179	1.0	0.02

Supplementary Table S2 ROC curve analysis for individual biomarkers

Metabolite	AUC	T-tests	Log_2FC	Cutoff *	Sensitivity	Specificity
9-cis-Retinoic acid	0.888	0.0047	1.385	-0.0709	0.8	0.9
Dehydrophytosphingosine	0.860	0.073	0.399	-0.197	0.8	0.9
LysoPC(16:1(9Z))	0.777	6..E-4	-1.078	0.129	0.9	0.6
LysoPC(18:3(9Z,12Z,15Z))	0.754	5.7E-3	-3.22	0.614	0.7	0.7
LysoPC(P-18:1(9Z))	0.688	0.012	1.185	-0.336	0.6	0.7
LysoPC(20:3(5Z,8Z,11Z))	0.671	0.081	-1.085	-0.0784/-0.0251	0.6/0.7	0.7/0.6
LysoPC(17:0)	0.624	0.109	-1.348	0.173	0.6	0.6
LPA(18:2(9Z,12Z)/0:0)	0.590	0.281	-1.026	0.123/0.161	0.5/0.6	0.6/0.5
LysoPC(22:6(4Z,7Z,10Z,13Z,16Z,19Z))	0.579	0.192	-1.380	-0.00189	0.4	0.8
LysoPC(20:4(5Z,8Z,11Z,14Z))	0.564	0.744	-0.744	-0.188	0.6	0.5
1H-Indole-3-carboxaldehyde	0.560	0.012	-2.498	0.106	0.4	0.9
LysoPC(18:2(9Z,12Z))	0.522	0.960	-0.655	-0.213	0.5	0.5
LysoPC(P-18:1(9Z))	0.511	0.879	-0.689	-0.107	0.8	0.4

*After log transformation
